# Impact of Climatic Factors on the Incidence of Cutaneous Leishmaniasis in Essaouira, Morocco: A Decadal Analysis (2014–2023)

**DOI:** 10.3390/epidemiologia7010028

**Published:** 2026-02-14

**Authors:** Said Benkhira, Najma Boudebouch, Bouchra Benazzouz

**Affiliations:** 1 Laboratory of Biology and Health, Ibn Tofail University, Kenitra 14000, Morocco; najma_boudebouch@yahoo.fr (N.B.); bouchra.benazzouz@uit.ac.ma (B.B.); 2 Higher Institute of Nursing and Technical Health Professions, Annex Essaouira, Ministry of Health and Social Protection, Marrakech 44000, Morocco

**Keywords:** cutaneous leishmaniasis, climatic factors, epidemiology, parasitic diseases, statistical correlation

## Abstract

**Background/Objectives**: Cutaneous leishmaniasis (CL) remains a major public health and economic challenge in Morocco, where its transmission dynamics are increasingly influenced by climatic variability. This study aimed to evaluate the impact of meteorological factors on CL incidence in the province of Essaouira, a high-incidence region, to identify the environmental drivers behind recent epidemic trends. **Methods**: Epidemiological data (N = 834 cases) were collected from the Hygiene and Health Laboratory of Essaouira for the period between January 2014 and December 2023. Climatic variables were obtained from the Moroccan Directorate of National Meteorology. Data were analyzed at annual, seasonal, and monthly scales using the Spearman rank correlation in R 4.5.0 software to account for non-normal distributions and non-linear associations. **Results**: CL incidence remained stable from 2014 to 2021 before an unprecedented surge in cases during 2022–2023. Annual analysis indicated that warm and dry years pose a higher risk, with incidence positively correlated with temperatures and negatively associated with humidity and precipitation. Monthly results identified a biphasic regulatory mechanism: a winter hydric constraint phase with strong negative correlations with January rainfall and humidity (*p* < 0.05), followed by a summer thermal promotion phase where minimum temperature (T_min_) emerged as the dominant driver (rho = 0.53), peaking in September (rho = 0.59). **Conclusions**: Our findings confirm the significant influence of climatic factors on CL incidence through complex seasonal dynamics. These results highlight the necessity of integrating high-resolution meteorological monitoring and predictive modeling into public health surveillance to anticipate future outbreaks in the context of increasing Mediterranean aridification.

## 1. Introduction

CL is a neglected tropical disease caused by protozoan parasites of the genus *Leishmania* and transmitted to humans through the bite of infected female phlebotomine sandflies [1,2,3]. Depending on the *Leishmania* species and the host’s immune response, the disease can cause three main clinical manifestations: localized CL, characterized by cutaneous ulcers, sometimes accompanied by satellite lesions and/ or nodular lymphangitis; muco-cutaneous leishmaniasis (MCL), involving the destruction of mucosal and cartilaginous tissues; and visceral leishmaniasis (VL), a potentially fatal form affecting internal organs such as the liver, spleen, and bone marrow [4]. CL represents a formidable public health and social challenge in tropical, subtropical, and Mediterranean regions [5]. It is currently endemic in nearly 100 countries, with an estimated 350 million people at risk [4], and 0.7 to 1.0 million new infections reported annually [6,7]. While most infections remain subclinical, a significant portion develops into symptomatic disease, resulting in 20,000–30,000 deaths each year [8].

The geographical footprint of leishmaniasis has expanded significantly in recent years, with over 90% of global CL cases concentrated in seven nations: Algeria, Afghanistan, Brazil, Iran, Peru, Syria, and Saudi Arabia [8,9]. Despite its wide distribution, it remains one of the world’s most neglected diseases, predominantly affecting impoverished populations in developing nations [10]. Morocco is a key endemic focus within the Mediterranean basin, where disease transmission is inextricably linked to specific climatic and ecological determinants [11]. Vector-borne diseases like CL are exceptionally sensitive to climate change because they rely on complex biological cycles involving multiple species of pathogens, vectors, and hosts [12]. Consequently, shifts in climatic patterns are recognized as key environmental drivers that may influence disease distribution, vector density, and seasonality, potentially facilitating the adaptation of transmission cycles to new environments [13,14,15,16].

Morocco, due to its geographical position in northwestern Africa, is particularly vulnerable to the impacts of climate change. National studies indicate that over the past 45 years, humid and sub-humid areas have diminished, giving way to semi-arid and arid regions. This shift is accompanied by an increase in the average annual temperature of 0.16 °C per decade and a 47% reduction in spring precipitation nationwide. Globally, data from the World Meteorological Organization (WMO) reveal that the past eight years have been the warmest on record, with 2016, 2019, and 2020 leading. In 2022, the global average temperature exceeded pre-industrial levels by 1.15 °C. In Morocco, that same year was the hottest in over 40 years, with a temperature anomaly of +1.63 °C compared to the climatological average of 1981–2010 [17].

Essaouira province, situated in the semi-arid region of southwestern Morocco, has reflected these broader environmental trends, experiencing notable increases in both leishmaniasis incidence and climatic volatility over the last decade [2,18].

Between 2014 and 2023, 834 cases of CL were diagnosed in the province, with the number of affected municipalities rising from 7 (12%) to 25 (44%). This period culminated in an unprecedented surge in 2023, which accounted for 22% of all cases in the decade, as the annual incidence rate rose from 11.1 per 100,000 inhabitants in 2015 to 40.3 per 100,000 in 2023 [2]. Despite the clear temporal association between these trends, localized studies investigating the precise correlation between climatic variables and CL incidence in Essaouira remain scarce. Therefore, this study aims to investigate the relationship between climatic drivers and the incidence of cutaneous leishmaniasis in Essaouira over the past ten years. By identifying the specific ecological dynamics at play, this research seeks to provide evidence-based insights to inform regional public health strategies for disease prevention and control.

## 2. Materials and Methods

### 2.1. Study Design and Setting

This retrospective observational study was designed to analyze the influence of climatic factors on the incidence of CL in the province of Essaouira, Morocco. The study period spanned ten years, from 1 January 2014 to 31 December 2023. Essaouira province is located within the Marrakech-Safi region and covers a total area of 6355 km^2^ [19]. It is geographically bounded by Safi province to the north, Agadir prefecture to the south, Chichaoua province to the east, and the Atlantic Ocean to the west. Administratively, the province comprises 57 communes (5 municipalities and 52 rural communes), which were grouped into 11 health constituencies according to the 2014 health division [2].

The region is characterized by a semi-arid climate, generally hot and dry throughout the year [18,20], influenced by both the Atlantic Ocean and the High Atlas Mountains, leading to significant seasonal variations [21]. The climate features an annual average temperature of 17.8 °C, with highs up to 40 °C and lows down to 2.2 °C. Precipitation is low and irregular, averaging 279 mm annually, with most rainfall occurring between November and April, and typically dry summers [21].

### 2.2. Data Sources and Variables

Epidemiological data, consisting of the monthly number of confirmed CL cases, were obtained from the official health records of the Hygiene and Health Laboratory within the Health Delegation of Essaouira. A total of 834 positive CL cases diagnosed during the study period were included, representing a complete enumeration of all recorded cases in the province. Climatic variables were sourced from the Moroccan General Directorate of Meteorology and included monthly mean temperature (°C), (T_min_), maximum temperature (T_max_), relative humidity (%), wind speed (m/s), and total rainfall (mm).

The primary outcome variable was the monthly incidence of CL, while the exposure variables were the aforementioned meteorological parameters. As this was a retrospective study relying on existing health records, efforts were made to use consistently collected data to minimize potential selection and information biases. No missing data were encountered for either the epidemiological or climatic variables during the study period.

### 2.3. Statistical Methods

Data pertaining to CL incidence and climatic factors were organized using Microsoft Excel (version 2016). All statistical analyses and visualizations were performed using RStudio (version 2025.5.0.0) with R software (version 4.5.0), employing specialized packages such as ggplot2, dplyr, and corrplot. Statistical significance was assessed using *p*-values for correlation coefficients at thresholds of *p* < 0.05, *p* < 0.01, and *p* < 0.001.

To evaluate the relationship between disease incidence and climatic drivers, we utilized Spearman’s rank correlation coefficient (rho). This non-parametric approach was selected over Pearson’s correlation due to the non-normal distribution of the annual incidence data, as confirmed by the Shapiro–Wilk test (W = 0.745; *p* = 0.003). Furthermore, Spearman’s coefficient is more robust in capturing non-linear monotonic relationships and handling the extreme incidence peaks observed in 2022 and 2023.

Data visualization included trend graphs, annual scatter plots, and monthly correlation heatmaps to identify temporal windows of risk. To assess the seasonal influence of climatic drivers, monthly data were aggregated into four distinct periods: Winter (December–February), Spring (March–May), Summer (June–August), and Autumn (September–November). As this study represents a complete enumeration of all diagnosed CL cases in the province rather than a sample, standard correlation and descriptive analyses were deemed appropriate without the need for sampling weights or design-based adjustments.

## 3. Results

### 3.1. Epidemiological Overview and Annual Temporal Trends

**Figure 1:**
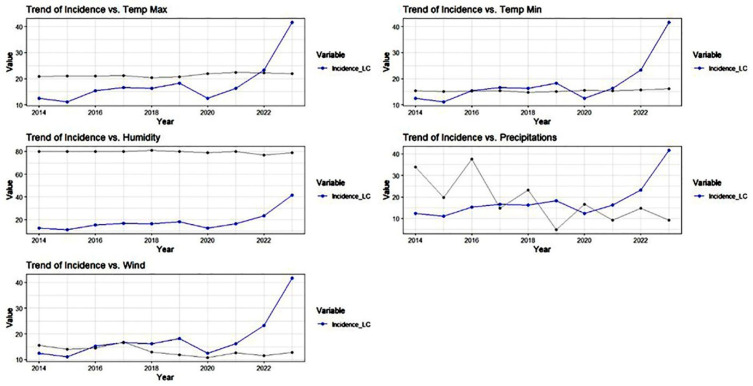
Annual trends of CL incidence and parallel evolution of climatic factors from 2014 to 2023. The black line represents the annual mean of the climatic factor specified in the title of each plot (Temp Max, Temp Min, Humidity, Precipitation, or Wind).

A total of 834 confirmed cases of CL were recorded by the Hygiene and Health Laboratory of Essaouira between 1 January 2014 and 31 December 2023. The annual incidence of CL over this ten-year period revealed two distinct epidemiological phases. From 2014 to 2021, the incidence remained relatively low and stable, fluctuating marginally around a baseline endemicity without a pronounced ascending or descending trend. However, the year 2023 marked a significant departure from this stability, characterized by a dramatic and unprecedented surge in cases that represented the highest epidemiological peak of the decade (Figure 1).

Interestingly, this massive outbreak in 2023 did not coincide with drastic variations in annual average climatic factors. The annual curves for T_max_, T_min_, humidity, and wind speed remained relatively stable or exhibited only minor fluctuations during the peak year. Similarly, while precipitation showed typical inter-annual variability, no exceptional hydrological pattern was observed in 2023 that could directly explain the explosion of CL cases at an annual resolution.

### 3.2. Global Annual Correlations

**Figure 2:**
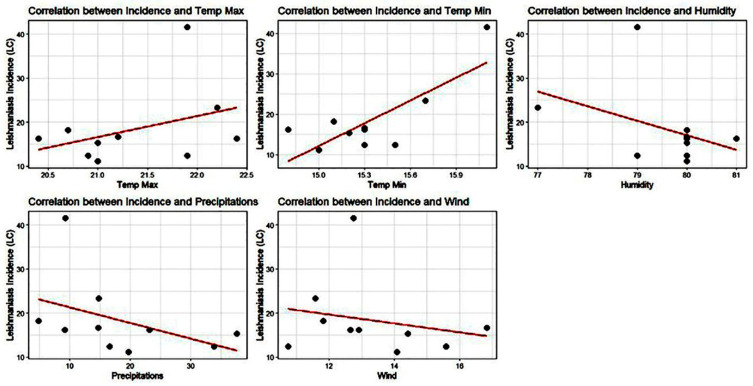
Annual correlations between CL incidence and meteorological variables. Each black dot represents a single observation year (annual mean of the climatic factor vs. total incidence), while the red line illustrates the corresponding linear regression trend.

The analysis of annual correlations between CL incidence and climatic drivers provided a global overview of environmental favorability (Figure 2). Spearman rank correlation indicated that years characterized by higher temperatures—particularly T_min_—and lower humidity and precipitation tended to have higher disease incidence. Specifically, annual T_min_ showed a more pronounced and consistent positive association with incidence compared to T_max_. Conversely, both humidity and precipitation exhibited negative annual correlations, suggesting that warmer and drier years create a higher risk for CL transmission. While wind speed showed a slightly negative annual correlation, the relationship appeared less robust than those identified for thermal and hydric factors. Despite these general trends, several outliers were noted, such as years with high incidence despite relatively high humidity, indicating that annual averages may mask the more granular dynamics occurring at monthly scales.

### 3.3. Monthly and Seasonal Dynamics: The Biphasic Regulatory Model

**Figure 3:**
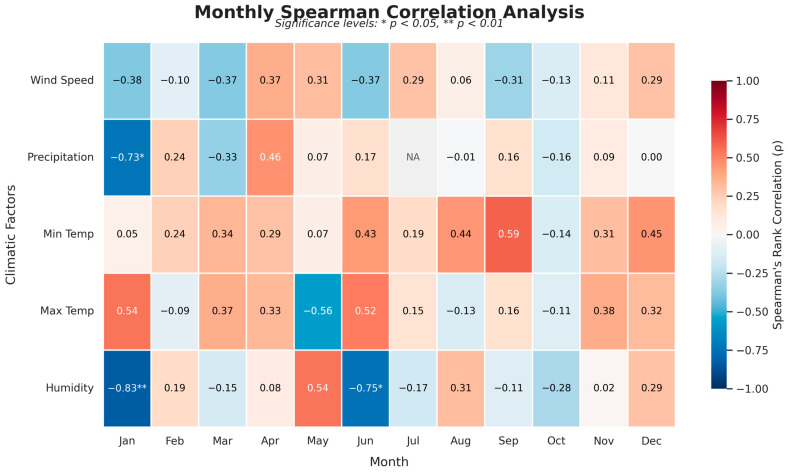
Heatmap of Spearman correlation coefficients between monthly CL incidence and climatic factors. The Not Available (NA) value for precipitation in July is due to zero variance resulting from a lack of rainfall during the study period.

To capture the complexity of the transmission cycle, a monthly disaggregation of climatic influences was performed (Figure 3). The results revealed a highly specific temporal regulation of CL incidence. Wind speed exhibited the most unstable correlations, though predominantly negative values in January (rho = −0.38) and June (rho = −0.37) suggest that calm atmospheric conditions may favor vector activity. Precipitation emerged as a major hydrological regulator during the winter, with a statistically significant negative correlation in January (rho = −0.73, *p* < 0.05). This indicates that rainfall deficits during the winter “priming” phase are primary drivers for subsequent increases in incidence.

Relative humidity emerged as the most consistent negative correlate, acting as a primary indicator of dry air stress. The strongest and most statistically significant relationship in the entire study was the negative correlation between January humidity and CL incidence (rho = −0.83, *p* < 0.01). A second significant negative peak in humidity was identified in June (rho = −0.75, *p* < 0.05), serving as a pre-summer alert for upcoming surges.

Thermal drivers showed a distinct seasonal shift. While T_max_ showed a positive correlation in January (rho = 0.54) followed by a transient reversal in May (rho = −0.56), T_min_ served as the consistent nocturnal thermal engine. The correlation between T_min_ and incidence increased progressively from June through August, peaking in September (rho = 0.59). Although these individual monthly thermal correlations did not reach formal statistical significance, their sustained high values over four consecutive months confirm that nocturnal warmth is a critical catalyst for maintaining the transmission cycle into the autumn.

**Figure 4:**
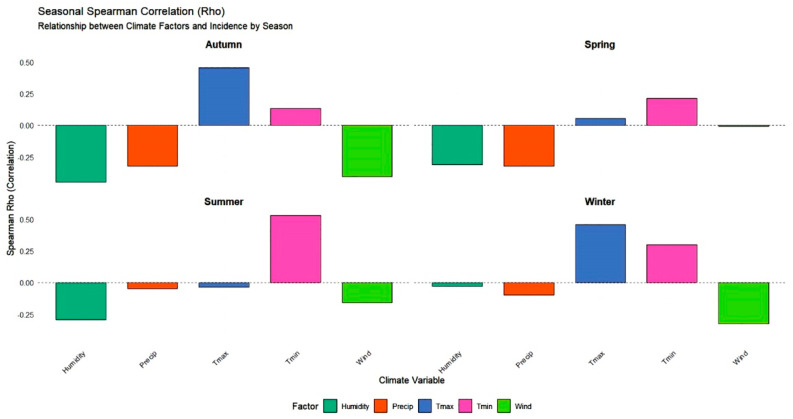
Seasonal Spearman Correlation (Rho) between Climatic Factors and Disease Incidence (2014–2023).

Seasonal aggregation (Figure 4) highlights the global climatic dominants that characterize different periods of the year, providing a broader perspective on the disease’s environmental drivers. During the summer, T_min_ emerges as the most influential factor (0.53), a predominance that is visually supported by bar chart analysis compared to other variables during this season. In contrast, winter dynamics are primarily marked by T_max_ (0.46) and wind speed (−0.32), while autumn is characterized by moderate negative correlations with both humidity (−0.43) and wind (−0.42). Notably, most correlations at this seasonal scale are classified as non-significant. This suggests a dilution effect, where the strong, specific signals observed at the monthly level are weakened during quarterly aggregation, indicating that climatic impacts on incidence are more precisely captured at a higher temporal resolution.

## 4. Discussion

This study aimed to examine the incidence dynamics of CL in relation to various climatic factors over a ten-year period (2014–2023). In line with this objective, our results revealed complex temporal trends and significant correlations between CL incidence and several climatic variables, including T_min_, humidity, and precipitation, at the annual, seasonal, and monthly scales. These observations provide valuable insights for a better epidemiological understanding of this vector-borne disease, highlighting the complex interaction between climatic and non-climatic elements influencing its dynamics.

A comparison across scales reveals a dilution effect of statistical signals during seasonal aggregation, proving that incidence responds to specific monthly windows of opportunity rather than smoothed quarterly averages. The incidence in our study area appears to follow a biphasic model: a winter priming phase limited by excessive precipitation, followed by a summer “amplification phase” regulated by optimal nocturnal temperatures and calm anemometric conditions

On an annual scale, T_min_ shows a notable positive correlation with the incidence of CL, which is explained by the fact that milder temperatures extend the life cycle and activity of phlebotomine sand flies, the insect vectors. These vectors, unlike mosquitoes, do not have an aquatic life stage but depend on humidity and temperature for their development, which confines them to tropical and subtropical regions where the temperature exceeds 15.6 °C for at least three months a year [22]. Active year-round in the tropics and during the summer in temperate zones, phlebotomine sand flies require humid biotopes rich in organic matter, such as rodent burrows or rock crevices, for the development of their larvae [23]. Conversely, annual humidity and precipitation are negatively correlated with incidence, suggesting that overall drier years would favor transmission, as excessive rainfall can flood larval breeding sites and reduce vector activity [24,25]. However, this relationship is nuanced, as heavy rainfall in the autumn and winter, followed by a dry spring and summer, can promote vegetation and, consequently, the proliferation of rodents and their associated phlebotomine sand flies, thereby creating favorable conditions for a high incidence of the disease. This observation is consistent with longitudinal studies conducted in Algeria (notably in Chott Ech Chergu), where it was found that the period of low disease incidence (1990–1999) coincided with prolonged droughts from May to November and humid conditions from December to March. In contrast, the period of high incidence (2000–2009), which saw annual peaks reaching 38 cases per 1000 inhabitants, was characterized by very rainy autumns and winters followed by relatively dry springs and extremely dry summers [26].

The correlation analysis of Spearman ranks at the monthly (Figure 3) and seasonal (Figure 4) scale reveals a complex dynamic for cutaneous leishmaniasis (CL), characterized by a duality of climatic constraints that exert complementary influences throughout the year. The most compelling finding is the identification of a decisive winter hydrological signal, where disease incidence exhibits a strong and statistically significant negative correlation with January humidity (−0.83; *p* < 0.01) and precipitation (−0.73; *p* < 0.05). This is consistent with research conducted in Senegal and Ivory Coast, which suggests that rainy winters reduce incidence, potentially by negatively affecting immature vector populations [27,28]. Furthermore, Chavy et al., in their study of Neotropical moist forest biome (including the the Amazon basin and French Guiana), highlighted the non-linear effect of rainfall: while cases may slightly increase at the onset of the rainy season, they decline sharply when precipitation becomes excessive [29], a trend that corroborates our correlation plots. The influence of dry air reappears significantly in June (−0.75; *p* < 0.05), aligning with observations from Ethiopia where increased humidity often associated with heavy rainfalls linked to a decrease in vector density [30].

Concurrently with this hydrological regulation, a thermal mechanism operates during the summer activity period. As sandflies are thermophilic insects, they require specific thermal ranges for survival and metabolic activity [25]. Our results demonstrate a progressive increase in the influence of T_min_ from June through September, peaking at (0.59). This trend is further supported by our seasonal analysis, where T_min_ emerges as the dominant factor during the summer (0.53), matching evidence that infestation rates increase proportionally with heat [31,32]. It is established that Eastern Mediterranean sandflies exhibit a metabolic growth peak around 28 °C [33], with optimal female abundance observed between 26 °C and 28 °C [34]. However, beyond these physiological thresholds, excessively high temperatures can lead to mass mortality [31,32]. The persistence of a positive correlation in December (0.45) suggests that mild nocturnal temperatures at the end of the year may prolong vector activity, confirming that these insects can maintain significant transmission potential until the late rainy season [35].

Finally, wind speed and temporal resolution complete this epidemiological framework. We observed a moderate negative correlation between wind speed and incidence, particularly in winter (−0.32) and during transition months such as March (−0.37) and June (−0.37). These findings align with data from Turkey showing that sandfly abundance is inversely related to wind velocity [36], with activity peaking during calm atmospheric conditions [37].

The analysis of factors influencing the incidence of CL reveals a complex interaction between climatic and non-climatic elements, the dynamics of which vary according to the time scale considered. The 2023 epidemic, for example, could be largely attributed to non-climatic factors such as changes in land use (deforestation, urbanization), population movements, or failures in vector control programs [38,39]. Furthermore, the relationships between climatic variables and vector-borne diseases are often non-linear, with outbreaks frequently associated with climatic extremes, when health systems and populations are ill-prepared [40].

Limits of the Study:

- The use of aggregated data at the Essaouira region level may mask significant local variations in climatic conditions and CL incidence.
- Correlations do not prove causation; other factors not measured in this study may influence the observed relationship.
- The 10-year study period, while providing a good overview of trends, could be extended to capture longer climatic or epidemiological cycles.
- Climatic data aggregated monthly or annually may not capture the complexity of daily or weekly weather events that directly influence vector dynamics.

## 5. Conclusions

This study highlights a significant shift in the epidemiological trajectory of cutaneous leishmaniasis in Essaouira province, characterized by a transition from relative stability to an unprecedented surge in cases during the 2022–2023 period. Our analysis identifies a biphasic climatic regulation: a winter priming phase driven by precipitation and humidity deficits, followed by a summer amplification phase governed by optimal nocturnal temperatures. Specifically, T_min_ emerged as the most consistent thermal engine for disease maintenance, while extreme aridity in January and June served as the primary hydrological drivers.

The significance of these findings lies in their ability to provide a localized environmental framework for understanding epidemic peaks in the Mediterranean basin, offering public health authorities specific monthly windows of risk. However, several limitations must be acknowledged. First, as this study relies on Spearman’s rank correlations, the associations identified do not imply direct biological causation but rather environmental favorability. Furthermore, other influential factors, such as vector-host dynamics, socio-economic shifts, and changes in urbanization, were not integrated into this climatic model.

Looking forward, future research should focus on the development of multi-variable predictive models and early warning systems that integrate real-time meteorological data. Strengthening the resilience of public health strategies will require a deeper integration of environmental monitoring to anticipate outbreaks. Ultimately, as the Mediterranean region faces increasing aridification, understanding the nuanced climatic drivers of leishmaniasis remains a critical priority for mitigating the burden of climate-sensitive diseases in North Africa.

## Data Availability

The datasets used and/or analyzed during the current study are not publicly available due to administrative restrictions and data confidentiality but are available from the corresponding author upon reasonable request.
